# Are the MORECare guidelines on reporting of attrition in palliative care research populations appropriate? A systematic review and meta-analysis of randomised controlled trials

**DOI:** 10.1186/s12904-019-0506-6

**Published:** 2020-01-09

**Authors:** Anna Oriani, Lesley Dunleavy, Paul Sharples, Guillermo Perez Algorta, Nancy J. Preston

**Affiliations:** 1 Palliative and Supportive Care Clinic, Oncology Institute of Southern Switzerland, via Ospedale 1, 6500 Bellinzona, Ticino Switzerland; 2 International Observatory on the End-of-Life Care, Lancaster University, Lancaster, UK; 3 Mathematics, International Observatory on the End-of-Life Care, Lancaster University, Lancaster, UK; 4 Division of Health Research, Lancaster University, Lancaster, UK; 5 International Observatory on the End-of-Life Care, Lancaster University, Lancaster, UK

**Keywords:** Randomised controlled trials, attrition, missing data, palliative care, systematic review, MORECare guidelines

## Abstract

**Background:**

Palliative care trials have higher rates of attrition. The MORECare guidance recommends applying classifications of attrition to report attrition to help interpret trial results. The guidance separates attrition into three categories: attrition due to death, illness or at random. The aim of our study is to apply the MORECare classifications on reported attrition rates in trials.

**Methods:**

A systematic review was conducted and attrition classifications retrospectively applied. Four databases, EMBASE; Medline, CINHAL and PsychINFO, were searched for randomised controlled trials of palliative care populations from 01.01.2010 to 08.10.2016. This systematic review is part of a larger review looking at recruitment to randomised controlled trials in palliative care, from January 1990 to early October 2016. We ran random-effect models with and without moderators and descriptive statistics to calculate rates of missing data.

**Results:**

One hundred nineteen trials showed a total attrition of 29% (95% CI 28 to 30%). We applied the MORECare classifications of attrition to the 91 papers that contained sufficient information. The main reason for attrition was attrition due to death with a weighted mean of 31.6% (SD 27.4) of attrition cases. Attrition due to illness was cited as the reason for 17.6% (SD 24.5) of participants. In 50.8% (SD 26.5) of cases, the attrition was at random. We did not observe significant differences in missing data between total attrition in non-cancer patients (26%; 95% CI 18–34%) and cancer patients (24%; 95% CI 20–29%). There was significantly more missing data in outpatients (29%; 95% CI 22–36%) than inpatients (16%; 95% CI 10–23%). We noted increased attrition in trials with longer durations.

**Conclusion:**

Reporting the cause of attrition is useful in helping to understand trial results. Prospective reporting using the MORECare classifications should improve our understanding of future trials.

## Background

Attrition is a major concern for accurate analysis of all trials and can influence the results of a study through potentially biasing the treatment effects and reducing the ability to detect differences [1–3]. Furthermore, conducting research with palliative care patients can be particularly challenging because of high levels of missing data and/or attrition due to high mortality rates and symptom burden [1, 4].

Authors report that the most important thing is to understand the reason for the missing data [5, 6]. Generally, missing data can be classified into three categories (Table 1): completely missing at random (CMAR), missing at random (MAR) and missing not at-random (MNAR), but in palliative care populations, missing data could mostly likely to be classified as MNAR because the patients being too unwell to complete a trial [1]. This is likely to be as a result of health deterioration, comorbidities and frailty [6], which are not random events [1, 7]. Recently, within the MORECare guidance, authors proposed three new categories to define the type of attrition in palliative care: attrition due to death (ADD), attrition due to illness (ADI) and attrition at random (AAR) [1]. Moreover, in 2013 the MORECare team developed guidance for conducting research with palliative care populations [8] and part of the checklist of conducting studies in palliative care, was how to deal with missing data and attrition [1, 8]. Consequently, the MORECare statements have been included in the EQUATOR Network website and database (http://www.equator-network.org/reporting-guidelines/morecare-statement/), to set clear standards on good practice in evaluating clinical studies in end of life care [9]. In fact, a barrier to the development of good practice in palliative care is the lack of quality research and evidence [8]. For this reason the MORECare guidance has been developed to identify research standards to aid future studies [8]. Furthermore, the inclusion of the MORECare guidance in the EQUATOR Network website could help to enhance the use of this guidance to improve the reliability of research in palliative care.

**Table 1:** Cathegories of missing data [1]

General classification	Definition
Completely missing at random (CMAR)	when missingness may depend on single variables
Missing at random (MAR)	when missingness is not related to the specified variables
Missing not at random (MNAR)	when missingness is related to a specific variable

A crucial aspect of clinical trials is the proportion of missing data and how researchers approach this to avoid serious biases [10]. The proportion of missing data is directly related to the quality of statistical inferences that can be made. Standard trial guidance suggests that the levels of missing data should be between 5 and 20%, meanwhile previous palliative care research has shown levels of missing data up to 80% of the total results [11]. Hussain’s (2016) review of the amount of missing data in clinical trials relating to palliative care populations found missing data levels of over 20% in half of the studies with an overall rate of 23.1%. In a detailed investigation of missing data in cancer trials involving palliative care populations Hui et al. (2013) found an attrition rate of 26% for the primary endpoint and 44% for participants reaching the end of the study [12]. Hui et al. also concluded that some investigators struggled to attribute the cause of the missing data.

Once the possible reasons for missing data are determined, the next step is to decide how to deal with them [6]. The method used to estimate the missing data needs to be reported, since different methods of estimating missing data, based on different assumptions, could lead to different conclusions [7]. For this reason, some literature suggests using more than one method for analysis and to discuss the potential bias of missing data [7]. This is particularly important for trials conducted in the field of palliative care, where most of the missing data are MNAR that could be estimated and minimised through the study design and taken account of in the final analysis. Moreover, there are no specific statistical methods recommended to analyise missing data not at random [13].

General guidance on the management of missing data in palliative care studies stated that missing data and attrition should be expected in a palliative care population and low levels of missing data or attrition could lead you to question whether the population was infact a palliative care population [1]. Another issue in trial reporting is describing or even deciding who the total palliative care population is. Only by defining this group can all potential participants be screened for eligiblility. In many hospitals a large number of patients could be deemed eligible for a study but they are spread out across different departments and include both out and inpatients. It is unclear what the usual practice is for identifying and screening palliative care populations and whether, for example, this means screening patients from particular outpatient clinics or carrying out database searches of diagnoses or symptoms [6].

Overall when reporting trials including palliative care populations three main areas were seen as requiring more scrutiny: classification of attrition, levels of attrition and their accompanying imputation methods and descriptions of trial populations.

In this study we wanted to investigate whether the MORECare classifications on reporting attrition in trials can be retrospectively applied to data retrieved from a systematic review on attrition in palliative care and thus help to better understand the reported results.

## Methods

Primary Aims:

- To describe whether the MORECare attrition classifications could be retrospectively applied to palliative care randomised controlled trials.
- To describe whether there were any statistical differences between cancer and non-cancer patients and between the enrolment settings.

Secondary Aims:

- To describe any methods used to handle missing data.
- To describe if there was any correlation between the length of the time to primary outcome measure and the total attrition rate.

We conducted a systematic review to identify randomised controlled trials (RCTs) conducted in the last 5 years in a palliative care field. This review followed the methods of a Cochrane review [14] . This systematic review is part of a larger review looking at recruitment to RCTs in palliative care which covers the period from January 1990 to early October 2016 [15]. From this larger review, we selected randomised controlled trials involving palliative care populations from the last 5 years as reporting was likely to be of a better standard.

### Identification and selection of studies

**Fig. 1:**
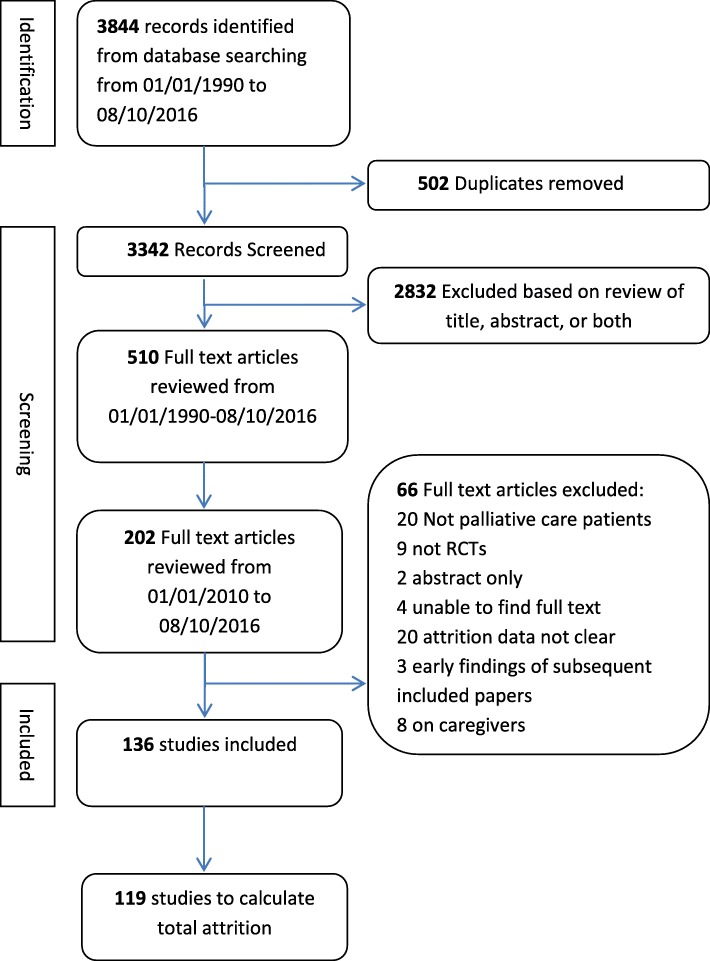
PRISMA study flow diagram

In the primary review [16] Embase, Medline, psychINFO and CINAHL databases were searched from the 1st January 1990 until the 8th October 2016 (see Table 2 and Fig. 1). Consequently, randomised controlled trials from 01.01.2010 to 08.10.2016, were extracted. The search included the terms palliat*, hospice* and ‘terminal care’ as they are seen as a robust and valid strategy to identify and retrieve palliative care literature [17–20]. The search terms used within Medline via EBSCO were palliat* or hospice* or terminal care or palliative care/ or palliative medicine/ or terminal care/ (not exploded) and randomi*ed. controlled trial* or randomised controlled trial/ (publication and topic). The search strategy was modified as necessary for the other databases searched (Table 2 for further details of the search terms used). The reference lists of the included studies were also hand searched to identify additional papers specifically focusing on recruitment to palliative care RCTs.

**Table 2:** Databases searched with search strategy

Electronic database	Search strategies
Medline via EBSCOhost	- palliat*
	- hospice*
	- terminal care
	- terminal care/ (not exploded)
	- palliative care/
	- palliative medicine/
	- randomi*ed. controlled trial*
	- randomised controlled trial/ (publication and topic)
	- limits: human, 01/01/2010 to 08/10/2016,Randomised Controlled Trials
PsycINFO via EBSCOhost	- palliat*
	- hospice*
	- terminal care
	- palliative care/
	- terminally ill patients/ - terminal cancer/
	- clinical trials/
	- randomi*ed. controlled trial*
	- limits 01/01/2010 to 08/10/2016, clinical trial, human.
CINHAL via EBSCOhost	- palliat*
	- hospice*
	- terminal care
	- palliative care/
	- terminal care/ (not exploded),
	- Randomi*ed. Controlled Trial*,
	- Clinical Trials/ (exploded),
	- randomised controlled trial/
	- limits 01/01/2010 to 08/10/2016, human and exclude Medline
Embase via Ovid	- palliat*
	- hospice*
	- terminal care
	- exp. palliative therapy/
	- terminal care/
	- randomi*ed. controlled*
	- randomized controlled trial/
	- limits human, RCTs, 01/01/2010 to 08/10/2016

### Study eligibility

Inclusion and exclusion criteria are listed in Table 3. AO and PS or LD screened the 3342 titles from the search. We used the WHO definition of palliative care which defines palliative care as an ‘approach which aims to improve the quality of life of patients and their families facing life threatening illness, through the prevention, assessment and treatment of pain and other problems, physical, psychosocial and spiritual’ [21] to identify palliative care populations.

**Table 3:**
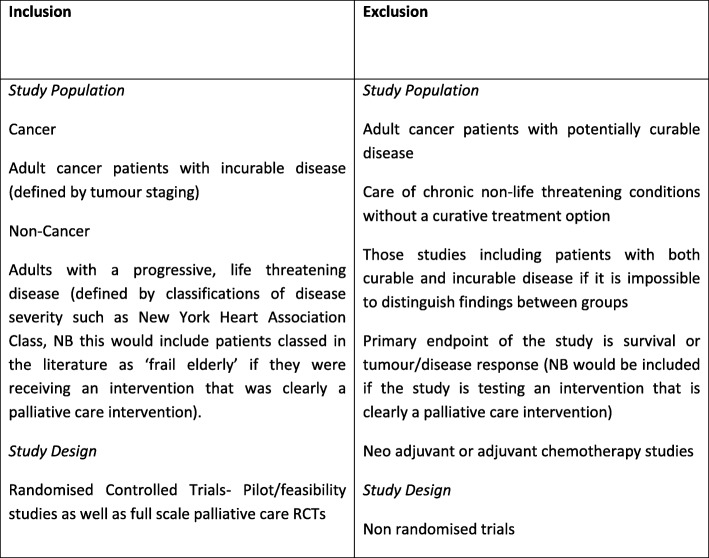
Inclusion and Exclusion Criteria

### Data extraction and analysis

Data were extracted by two independent reviewers (AO & PS or LD). If no agreement could be reached about inclusion of data extraction, an arbitrator (NP) was consulted. If there was insufficient information to make a decision about data, authors were contacted via email. If information were not forthcoming then the paper was excluded.

Data were collected to analyse the primary aim of the study, which was the retrospective application of the MORECare classifications on attributing the cause of attrition. We used the same criteria as Hussein (2016) to identify attrition, namely the number of participants lost by the time of the evaluation of the primary end point or final assessment if the primary endpoint was not made. The primary outcome was chosen because it was the most important outcome. Moreover it should have been defined at the time the study was designed to reduce bias [22] . In addition, we included attrition cases which occured between consent and randomization where available. Reasons for attrition were determined retrospectively to one of the MORECare classifications of ADD, ADI or AAR based upon decriptions within the paper. We used weighted means to describe the proportion of missing data.

To describe whether there were any statistical differences between cancer and non-cancer patients and between enrollment setting, we ran random effect models with and without moderators, using the Metafor package [23]. We aggregated double arcsine transformed values computing a weighted mean (with inverse-variance weight). To ease interpretation, we back transformed averages to estimated true proportions with corresponding 95% confidence intervals using Miller’s formula [24].

We used descriptive analysis to describe which imputation methods were used. The mean length of the time to primary outcome was calculated to assess whether length of stay was correlated with increased attrition rate using the Pearson correlation. Because the heterogeneity of data, it was not possible to calculate any correlation with patients’ overall survival, primary outcome and attrition.

### Quality assessment

The quality of the trial was not assessed as the focus of the review was on attrition rates as recorded in the study.

## Results

### Study selection

Of the 3342 titles and abstracts screened, full text articles of 202 studies were assessed for eligibility of which 136 were included in the final analysis, which included 17,472 participants (Table 4).

**Table 4:** characteristics of included studies

Lead author	Total attrition rate	Attrition rate -intervention arm	Attrition rate - control arm
Abernethy, A [25]	28/239 (11.7%)	8/120 (6.7%)	20/119 (16.8%)
Abernethy, A [26]	314/461 (68.1%)	69%	65.90%
Ahmedzai, S [27]	51/184 (27.7%)	26/92 (28.3%)	25/92 (27.2%)
Ando, M [28]	9/77 (11.7%)	4/38 (10.5%)	5/39 (12.8%)
Aoun, S [29]	15/58 (25.9%)	12/38 (31.6%)	3/20 (15%)
Barton, R [30]	11/22 (50%)	5/11 (45.5%)	6/11 (54.5%)
Bausewein, C [31]	34/70 (48.6%)	14/38 (36.8%)	20/32 (62.5%)
Beijer, S [32]	43/100 (43%)	22/51 (43.1%)	21/49 (42.9%)
Bennett, M [33]	5/24 (20.8%)	2/12 (16.7%)	3/12 (25%)
Bhatnagar, S [34]	NA	NA	NA
Brännström, M [35]	12/72 (16.7%)	NA	NA
Breitbart, W [36]	52/90 (57.8%)	24/49 (49%)	28/41 (68.3%)
Breitbart, W [37]	53/120 (44.2%)	31/64 (48.4%)	22/56 (39.2%)
Brisbois, T [38]	25/46 (54.3%)	13/24 (54.2%)	12/22 (54.5%)
Bruera, E [39]	49/190 (25.8%)	36/142 (25.4%)	13/48 (27.1%)
Bruera, E [40]	27/129 (20.9%)	14/63 (22.2%)	13/66 (19.7%)
Chan, C [41]	27%	11%	42%
Chen, H [42]	32/90 (35.6%)	14/45 (31.1%)	18/45 (40%)
Cheville, A [43]	10/66 (15.2%)	7/33 (21.2%)	3/33 (9.1%)
Chochinov, H M [44]	115/441 (26.1%)	57/165 (34.5%)	58/276 (21%)
Chow, E [45]	298/819 (36.4%)	148/406 (36.5%)	150/413 (36.3%)
Cruciani, R [46]	167/376 (44.4%)	85/189 (45%)	82/187 (43.9%)
De Raaf, P [47]	60/152 (38.2%)	40/76 (52.6%)	20/76 (26.3%)
Del Fabbro, E [48]	14/43 (32.6%)	6/19 (31.6%)	8/24 (33.3%)
Donovan, H [49]	17/65 (26.2%)	16/33 (48.5%)	1/32 (3.1%)
Edmonds, P [50]	6/52 (11.5%)	1/26 (3.8%)	5/26 (19.2%)
El-Jawahri, A [51]	0/50	0/23	0/27
Epstein, A [52]	2/56 (3.6%)	1/30 (3.3%)	1/26 (3.8%)
Farquhar, M [53]	20/67 (29.9%)	12/35 (34.3%)	8/32 (25%)
Fischer, S [54]	30/64 (46.9%)	14/32 (43.75%)	16/32 (50%)
Galbraith, S [55]	NA	NA	NA
Galfin,J [56]	9/34 (26.5%)	6/19 (31.6%)	3/15 (20%)
Gebbia, V [57]	10/86 (11.6%)	5/42 (11.9%)	5/39 (12.8%)
Greer, J [58]	12/40 (30%)	6/20 (30%)	6/30 (30%)
Gutgsell, K [59]	2/200 (1%)	1/100 (1%)	1/100 (1%)
Hardy,J [60]	113/187 (60.4%)	54/93 (58.1%)	57/92 (62%)
Heisler, M [61]	32/160 (20%)	16/84 (19%)	16/76 (21.1%)
Henke, C [62]	15/44 (34.1%)	6/24 (25%)	9/20 (45%)
Herr, K [63]	NA	NA	NA
Homsi, J [64]	5/37 (13.5%)	NA	NA
Hopkinson, J [65]	15/65 (23.1%)	10/35 (28.6%)	5/30 (16.7%)
Hui, D [66]	0/20	0/10	0/10
Hui, D [67]	7/30 (73.3%)	2/15 (13.3%)	5/15 (33.3%)
Ishiki, H [68]	6/27 (22.2%)	NA	NA
Israel, F [69]	9/31 (29%)	1/11 (9.1%)	8/20 (40%)
Johnson, J [70]	33/177 (18.6%)	25/118 (21.2%)	8/59 (13.6%)
Jones, L [71]	9/77 (11.7%)	3/42 (7.1%)	6/35 (17.1%)
Jones, L [72]	5/41 (12.2%)	1/21 (4.8%)	4/20 (20%)
Julião, M [73]	44/80 (55%)	22/39 (56.4%)	22/41 (53.7%)
Kerr, C [74]	4/34 (11.8%)	2/17 (11.8%)	2/17 (11.8%)
Kirste, S [75]	10/44 (22.7%)	7/22 (31.8%)	3/22 (13.6%)
Lee, C [76]	3/9 (33.3%)	2/4 (50%)	1/5 (20%)
Liao, J [77]	14/160 (8.75%)	9/66 (13.6%)	5/94 (5.3%)
Lim, J [78]	NA	NA	NA
Lloyd-Williams, M [79]	44/100 (44%)	24/49 (49%)	20/51 (39.2%)
López-Sendín, N [80]	9/24 (37.5%)	4/12 (33.3%)	5/12 (41.7%)
Lundholm, K [81]	9/31 (29%)	5/17 (29.4%)	4/14 (28.6%)
McLean, L [82]	6/42 (14.3%)	4/22 (18.2%)	2/20 (10%)
Mok, E [83]	26/84 (31%)	15/44 (34.1%)	11/40 (27.5%)
Ng, C [84]	49/88 (55.7%)	23/44 (52.3%)	26/44 (59.1%)
Oldervoll, L [85]	68/231 (29.4%)	43/121 (35.5%)	25/110 (22.7%)
Oxberry, S [86]	4/39 (10.3%)	NA	NA
Pantilat, S [87]	26/107 (24.2%)	13/54 (24.1%)	13/53 (24.5%)
Pelayo-Alvarez, M [88]	7/124 (5.6%)	3/66 (4.5%)	4/58 (6.9%)
Popa-Velea, O [89]	NA	NA	NA
Portenoy, R [90]	97/360 (26.9%)	72/269 (26.8%)	25/91 (27.5%)
Rhondali, W [91]	2/80 (2.5%)	NA	NA
Ringash, J [92]	NA	41%	53%
Salas, S [93]	0/20	0/11	0/9
Schofield, P [94]	29/108 (26.9%)	17/55 (30.9%)	12/53 (22.6%)
Sidebottom, A [95]	33/232 (14.2%)	23/116 (19.8%)	10/116 (8.6%)
Stein, R [96]	58/120 (48.3%)	24/55 (43.6%)	34/65 (52.3%)
Sternberg, C [97]	NA	NA	NA
Suh, S [98]	3/41 (7.3%)	2/20 (10%)	1/21 (4.8%)
Temel, J [99]	115/151 (76.2%)	NA	NA
Uitdehaag, M [100]	7/21 (33.3%)	3/11 (27.3%)	4/10 (40%)
Uitdehaag, M [101]	133/138 (96.4%)	67/70 (95.7%)	66/68 (97.1%)
Vogel, R [102]	6/35 (17.1%)	4/20 (20%)	2/15 (13.3%)
Volandes, A [103]	83/150 (55.3%)	40/70 (57.1%)	43/80 (53.75%)
Wallen, G [104]	102/152 (67.1%)	54/76 (71.1%)	48/76 (63.2%)
Wentlandt, K [105]	18/451 (4%)	NA	NA
Wyatt, G [106]	NA	NA	NA
Zaghloul, M [107]	11/40 (27.5%)	3/20 (15%)	8/20 (40%)
Zimmermann, C [108]	175/461 (38%)	97/228 (42.5%)	78/233 (33.5%)
Laltanpui, C [109]	NA	NA	NA
Litterini, A [110]	52/66 (78.8%)	23/34 (67.7%)	29/32 (90.6%)
Mariani, P [111]	13/80 (16%)	8/ 43 (18.6%)	5/37 (13.5%)
Ng, C [112]	0/60	0/30	0/30
Strong, R [113]	0/11	NA	NA
Vermandere, M [114]	6/55 (11%)	3/28 (10.7%)	3/27 (11%)
Agrwal, K [115]	5/49 (10.2%)	3/25 (12%)	2/24 (8.3%)
Ahmed, N [116]	30/182 (16.4%)	14/87 (16%)	16/95 (16.8%)
Anter, A [117]	12/100 (12%)	NA	NA
Badr, H [118]	1/39 (2.5%)	0/20	1/19 (5.3%)
Bajwah,S [119]	6/53 (11.3%)	3/26 (11.5%)	3/27 (11.1%)
Bakitas, M [120]	94/207 (45.4%)	45/104 (43.2%)	49/103 (47.5%)
Berwouts, D [121]	2/45 (4.4%)	2/30 (6.6%)	0/15
Buckingham, S [122]	13/32 (40.6%)	8/24 (33%)	5/8 (62%)
Chan, K [123]	20/29 (69%)	10/14 (71%)	10/15 (66%)
Currow, D [124]	19/106 (18%)	7/52 (13.4%)	12/54 (22.2%)
Davies, H [125]	10/106 (9.4%)	7/54 (13%)	3/52 (5.8%)
Eguchi,K [126]	1/35 (3%)	0/18	1/17 (6%)
Eldeeb, N [127]	NA	NA	NA
Fallon, M [128]	56/233 (24%)	32/116 (27.5%)	24/117 (20.5%)
Hardy, J [129]	NA	NA	NA
Higginson, I [130, 131]	23/105 (22%)	11/53 (20.7%)	12/52 (23%)
Higginson, I	6/52 (11.5%)	1/26 (3.8%)	5/26 (19.2%)
Hopp, F [132]	NA	NA	NA
Ibrahim, I [133]	1/39 (2.5%)	1/21 (5%)	0/28
Matlock, D [134]	10/51 (19.6%)	8/25 (32%)	2/26 (8%)
Jensen, W [135]	5/26 (19.2%)	3/13 (23%)	2/13 (15%)
Jacobs, C [136]	12/73 (16.4%)	6/38 (15.8%)	6/35 (17.1%)
Jatoi, A [137]	NA	NA	NA
Kwekkeboom, K [138]	8/86 (9.3%)	7/43 (16%)	1/43 (2.3%)
Li,F [139]	9/84 (10.7%)	2/28 (7.1%)	7/56 (12.5%)
Lund Rasmussen, C [140]	28/72 (38.8%)	13/34 (38.2%)	15/38 (39.4%)
Maddocks,M [141]	21/49 (42.8%)	15/30 (50%)	6/19 (31.5%)
Maltoni, M [142]	78/207 (37.7%)	35/100 (35%)	43/107 (40.1%)
McMillan, S [143]	366/716 (51%)	NA	NA
Nava, S [144]	11/200 (5.5%)	11/99 (11%)	0/101 (0%)
Nilssom,S [145]	23/100 (23%)	15/51 (29.4%)	8/49 (16.3%)
Okur,E [146]	1/48 (2%)	1/24 (4%)	0/24
Ozkul,S [147]	NA	NA	NA
Rief, H [148]	24/60 (40%)	12/30 (40%)	12/30 (40%)
Saha, A [149]	0/40	0/20	0/20
Sau,S [150]	NA	NA	NA
Steel,J [151]	140/261 (53.6%)	76/144 (53%)	64/117 (55%)
Wadhwa, D [152]	NA	NA	NA
Warth, M [153]	16/84 (19%)	4/42 (9.5%)	12/42 (28.5%)
Warth, M [154]	11/84 (13%)	3/42 (7.1%)	8/42 (19%)
Xue,D [155]	NA	NA	NA
Yousef, A [156]	0/120	0/60	0/60
Tarumi, Y [157]	18/74 (24.3%)	10/35 (28.6%)	8/39 (20.5%)
Mc Corke,R [158]	54/146 (37%)	30/66 (45.4%)	24/80 (30%)
Allen,R [159]	25/45 (55%)	16/22 (73%)	9/23 (39.1%)
Wong, F [160]	16/84 (19%)	6/43 (14%)	10/41 (24.3%)

### Study characteristics

We needed to decide which was the intervention and the control arm in 7 studies, because it was not specified by the authors. The median sample size was 75 (IQR 106). Among all the collected randomised controlled trials,few had a specific study design: 24 studies were feasibility/pilot studies, 3 were cluster trials, 2 were cross-over trials, one a fast-track trial. One study was designed to test the dose of a new drug. Four studies involved patients and their carers and one study patients and primary physicians. The median duration of studies to primary outcome measure was 7 weeks (IQR 11) with some studies having an intervention length of only a few hours or days. Thirteen studies did not mention the intervention duration.

In 5 studies participants were recruited from the hospice and in 28 from the hospital but it is unclear if these were inpatients and/or outpatients. The most common specific site mentioned in 47 studies was a ‘clinic’ which presumably meant outpatients. From the participants 25% were recruited from inpatient services, 30% from outpatient services and 16% recruited from both out patients and inpatients. For the remaining participants no indication was given as to whether they were inpatients or outpatients. Most patients had cancer (76%) with 20% having a non-cancer condition including heart failure, neurological conditions, respiratory, renal and liver disease or frail elderly populations. The remaining studies (4%) did not specify the patient’s condition.

### Application of MORECare classifications

The attrition rate was not recorded in 17 trials leaving 119 trials with assessable total attrition data. We presented the data only with descriptive statistics because there were not sufficient data to calculate rates of attrition in the ADD, ADI and AAR groups (Table 5).

**Table 5:** Weighted means attrition using MORECare criteria (*n* = 91)

Type of Attrition	Weighted Mean %	SD
Attrition due to Death	31.6	27.4
Attrition due to Illness	17.64	24.5
Attrition at random	50.8	26.5

We applied the MORECare classifications of attrition to 91 out of 119 papers that contained sufficient information on the cause of missing data. This reflects the difficulty in attributing the cause of missing data based upon the authors’ descriptions in the published papers. Some authors reported withdrawal as a cause of attrition, without specifying if this was related to a specific cause such as adverse events.

We found the main reason for attrition was attrition due to death (ADD) and accounted for a weighted mean of 31.6% (SD 27.4) of attrition cases. Attrition due to illness (ADI) was cited as the reason for 17.6% (SD 24.5) of participants. For 7% of total participants they left due to adverse events. In 50.8% (SD 26.5) of cases, the attrition was at random (AAR) with reasons such as patients being no longer contactable.

The weighted average attrition across all studies was 29% (95% CI 28–30%). The statistical analysis including participants’ diagnosis as a covariate (cancer vs non-cancer), was possibile in 113 studies. We did not observe significant differences between groups (non-cancer patients, 26%; 95% CI 18–34%; cancer patients, 24%; 95% CI 20–29%).

We were able to calculate whether including the study setting, inpatients and non-inpatients, as a covariate in 68 studies. We found significant differences between the two groups (*p* = 0.01), with a higher attrition rate for outpatients (29%; 95% CI 22–36%) than inpatients (16%; 95% CI 10–23%). These estimated proportions apperared to be all heterogeneous (*ps <* 0*.*0001). In some studies, authors did not distinguish the amount of inpatients for the amount of outpatients, thus it was not possible to conduct any statistical analysis.

Twenty trials reported data about attrition between enrolment and randomisation. These pre-randomisation data were too heterogeneous to be analysed. Although only 20 trials reported these missing data it may have been true for other studies too but not mentioned. Moreover, some authors commented upon the level of missing data in their papers, whilst in others no comment was made but attrition data was calculated from the CONSORT flow-chart. Because the data were heterogeneous, it was not possible to calculate any statistical difference between the studies that commented upon attrition and those studies that did not.

### Use of imputation methods for primary endpoint

According to primary endpoint, 74 of 136 studies (54%) commented that they used an imputation method for missing data but only 36 (26%) recorded how they managed their missing data (Table 6).

**Table 6:** imputation methods

Methods		Number of studies
Missing at random		4
Treated as separate category		1
Single Imputation	AUC analysis	2
	Last observed carried forward analysis	6
	Last observation carried forward based on intention to treat analysis	1
	Logistic regression method	1
	Regression model incorporating baseline covariates	1
	Mean imputation	1
	Conservative statistic (NMAR)	1
	Baseline group mean imputed as a null effect both for pre and post-intervention analysis	1
	Continuous time variable with random slope in a longitudinal model	1
	Monte Carlo error computation model including all the variables to be used in the analysis	1
Multiple imputation stathistical methods		15

As previously described, imputation methods should be reported, since different methods of estimating missing data, based on different assumption, could lead to different conclusions [7]. For this reason, part of literature suggests using more than one method for analysis and to discuss the potential bias of missing data [7]. Despite these recommendations, authors used different multiple imputation methods in only 15 studies. These methods were not uniform and different among each study.

Among the feasibility studies, one considered missing data as a random effect, five used a single imputation method (Area Under the Curve analysis, last observation carried forward, intention to treat analysis, conservative statistic). Only in one study, authors did not impute missing data because the main intention of the study was the feasibility of the intervention and also to explore the nature of missing data.

In few other studies [14], authors used different non statistical methods to deal with missing data, for example adapting their protocol to reduce the number of missing data (i.e. adapting the time of follow up or a specific questionnaire).

### Length of intervention

In 108 out of the 136 studies, it was possible to describe the length of the intervention. In the remaining studies this was not possible because it was not clearly reported by the authors. The median time to primary outcome measure was 7 weeks. There was a significant correlation (r = 0.37, *p* < 0.01) between the length of time to primary outcome measure and the total attrition rate, meaning that the longer the time to primary outcome the increased chance of attrition.

## Discussion

In this review we found that the MOREcare classifications could be applied retrospectively in about 67% of studies. In the remaining papers this was not possible due to insufficient details in reporting the reasons for missing data. We could not calculate any analysis in relation to the reason for attrition using the MORECare classifications due to insufficient data. Vague phrases such as withdrawn do not inform the reader as it is still unclear what the the reasons for the withdrawal were, for example was it due to progression of the illness or side effects of a drug or another reason? Dumville et al. (2006) recommend reporting the causes of attrition clearly to help understand the findings of a study [161] and applying the MORECare classifications gives an indication of not only what has happened in a trial but also the characteristics of the population involved.

Our review emphasises the need to identify primary outcome measures which should be measured sooner than later given the large amount of missing data in longer studies. Given the median time to primary endpoint was only 7 weeks, this shows that we are looking at end points potentially shorter than this but obviously this depends upon the focus of the study.

Palliative care populations are difficult to identify and these findings show a variation in where researchers looked for potential participants. Whilst we were able to make some comment upon where populations were identified from, this was difficult to extract as it was poorly recorded.

The level of missing data was higher than in other reviews (Hussain 2016; Hui 2013) which may reflect a broader definition of a palliative population. This is also reflected in the higher attrition rates noted in the non-cancer population and non-hospital populations. In the study by Hui (2013) the lower rates of attrition were in a cancer population based in one hospital. Modifications in trial design should be made for trials involving non-cancer, community based populations, as attrition rates were shown to be highest in these groups. Interestingly, we identified attrition even before randomisation. Perhaps this is something trial steering committees could monitor to assess the cause of attrition using the MORECare classifications, as it may help decide if attrition is due to the trial design or the population under study.

Only 26% of studies used any sort of imputation method for the primary outcome. All studies should comment upon missing data and notably report attrition following CONSORT guidelines not only for the primary outcome, but also for all the obtained results. Given all these studies were completed since 2010 you would expect this figure to be higher. With a rise in publishers asking for guidance in reporting of research to be followed hopefully this figure will increase. Moreover, according to the different type of missing data, different imputation methods can be used and it is recommended to use multiple imputation methods as a powerful tool for handling missing data with a sensitivity analysis [13].

One major concern about our review is that we relied upon our interpretation of descriptions of populations which we then decreed as palliative or not. Although the reviewers used the same definitions, their interpretation of the studies could have biased the reported results. Moreover, the causes of attrition have been interpreted according to the reasons given by the authors of the studies, which were not always clear. Hence some studies were excluded from this review because the causes of attrition were not clear. This may have changed the findings. As described, the high heterogeneity of collected data prevented further statistical analyses, such as calculation of the rates of attrition according to whether participants were in/outpatients, had cancer or not, or, according to type of attrition (ADD, ADI and AAR). The fact that most of the studies were about cancer patients limits the generalisability of our study in non-oncological settings. Moreover, most of the included studies were conducted in English-language nations.

This review included only randomised controlled trials, but more research is needed about the impact of missing data in other types of study design [162]. We assumed that from 2010, studies had a better standard of reporting and handling missing data. Further analysis about the correlation between the year of publication and the rate of missing data could have been assessed to analyse whether the reporting of missing data has improved over time.

## Conclusion

The MORECare classifications provided a useful tool in highlighting attrition due to death in a readily accessible manner. In particular higher rates of attrition should be expected in longer trials, non-cancer andcommunity based palliative care populations. By applying the MORECare classifications we should be able to characterise trial populations more easily to enable a better understanding of the trials results. Moreover, the use of these classifications may help the readers to understand if authors described clearly the rate of missing data and if authors tried to take into account the rate of attrition in the interpretation of their results. The MoreCare guidelines could also help researchers to better design and conduct their studies in palliative care settings. In fact, the difficult we had in the collection of the data shows that more efforts should be done to report the results of the studies and to handle with missing information that could potentially bias the final results.

## Data Availability

Not applicable. The research strategy and the list of the included articles, are in the manuscript file.

## Abbreviations

ADD: Attrition due to death
ADI: Attrition due to illness
AO: Anna Oriani
ARR: Attrition at random
CI: Interval of Confidence
CMAR: Completely missing at random
IQR: Interquartile Range
LD: Lesley Dunleavy
MAR: Missing at random
MNAR: Missing not at-random
NP: Nancy Preston
PS: Paul Sharples
RTCs: Randomised controlled trials
SD: Standard Deviation
